# Volatile Organic Compound Emissions from Different Stages of *Cananga odorata* Flower Development

**DOI:** 10.3390/molecules19078965

**Published:** 2014-06-27

**Authors:** Xiao-Wei Qin, Chao-Yun Hao, Shu-Zhen He, Gang Wu, Le-He Tan, Fei Xu, Rong-Suo Hu

**Affiliations:** 1 Spice and Beverage Research Institute, Chinese Academy of Tropical Agricultural Science (CATAS), Wanning, Hainan 571533, China; E-Mails: qin_xiaowei@163.com (X.-W.Q.); haochy79@163.com (C.-Y.H.); heshuzhen09@163.com (S.-Z.H.); wugang0225@sina.com (G.W.); xufei_0302054@163.com (F.X.); hnhrs@126.com (R.-S.H.); 2 Key Laboratory of Genetic Resources Utilization of Spice and Beverage Crops, Ministry of Agriculture, Wanning, Hainan 571533, China; 3 Hainan Provincial Key Laboratory of Genetic Improvement and Quality Regulation for Tropical Spice and Beverage Crops, Wanning, Hainan 571533, China

**Keywords:** *Cananga odorata*, volatile organic compound, flower development, HS-SPME-GC-MS

## Abstract

Headspace-solid phase microextraction-gas chromatography-mass spectrometry (HS-SPME-GC-MS) was used to identify the volatile organic compounds (VOCs) of the different flower development stages of *Cananga odorata* for the evaluation of floral volatile polymorphism as a basis to determine the best time of harvest. Electronic nose results, coupled with discriminant factor analysis, suggested that emitted odors varied in different *C. odorata* flower development stages, including the bud, display-petal, initial-flowering, full-flowering, end-flowering, wilted-flower, and dried flower stages. The first two discriminant factors explained 97.52% of total system variance. Ninety-two compounds were detected over the flower life, and the mean Bray–Curtis similarity value was 52.45% among different flower development stages. A high level of volatile polymorphism was observed during flower development. The VOCs were largely grouped as hydrocarbons, esters, alcohols, aldehydes, phenols, acids, ketones, and ethers, and the main compound was β-caryophyllene (15.05%–33.30%). Other identified compounds were β-cubebene, d-germacrene, benzyl benzoate, and α-cubebene. Moreover, large numbers of VOCs were detected at intermediate times of flower development, and more hydrocarbons, esters, and alcohols were identified in the full-flowering stage. The full-flowering stage may be the most suitable period for *C. odorata* flower harvest.

## 1. Introduction

*Cananga odorata* (Lam.) Hook. f. & Thomson, commonly known as ylang-ylang, belongs to the Annonaceae family and the *Cananga* genus. This perennial tropical tree, native to the Indonesian archipelago, is cultivated primarily in Comoros, Mayotte, and Madagascar. The tree is also grown in the Philippines, Reunion, Thailand, Vietnam, and China [1,2]. The aroma emitted by its flowers has caused ylang-ylang to be widely utilized for garlands, headdresses, and other personal adornments. Moreover, the heavily scented flowers are used in the cosmetics industry. In particular, ylang-ylang essential oil, obtained from fresh flowers of *C. odorata* through water steam distillation or chemical extraction, is one of the most important raw materials used in the perfume industry in a variety of applications, from high-grade perfume production to the soap industry. Moreover, this oil has applications in aromatherapy and is approved for food use by the US Food and Drug Administration [3,4]. Therefore, *C. odorata* has high economic and ornamental value because for its use as fragrance and food flavor properties.

Numerous studies have already been conducted to detect the odoriferous molecules released by *C. odorata* [5,6,7]. As reported in the literature, the identified volatile compounds in ylang-ylang oil are largely grouped as monoterpenes, terpenic alcohols, sesquiterpenic alcohols, sesquiterpene hydrocarbons, acetates, benzoates, and phenols [8,9]. Moreover, the major oil components are *p*-cresyl methyl ether, methyl benzoate, linalool, benzyl acetate and geranyl acetate, β-caryophyllene, d-germacrene, and (*E*,*E*)-α-farnesene [10]. According to the measurement and comparison of 15 major compounds of ylang-ylang oil from Comoros, Mayotte and Madagascar, the ISO 3063: 2004 (E) norm was published by the French standardization system (AFNOR, French standard) [11]. However, most studies on this subject have only focused on analyzing the volatile organic compounds (VOCs) emitted by ylang-ylang oil. Limited information is available on the floral volatile polymorphism of *C. odorata* at different flower development stages. Moreover, the volatile compounds of different flower development stages sever as indicators that determine the time of harvest, quality of essential oil, and flavor of food, among others. Therefore, this study aims to investigate the floral volatiles in *C. odorata* and to evaluate the volatile polymorphism of different flower development stages to determine the best time of harvest.

## 2. Results and Discussion

### 2.1. Discrimination of Different Flower Stages by Electronic Nose

**Figure 1:**
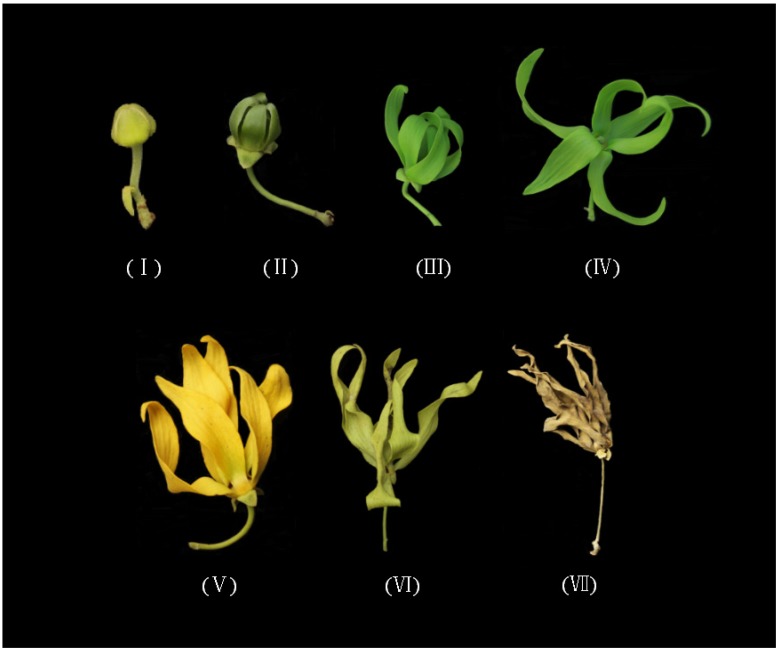
The morphological characteristics of *C. odorata* flower in seven different stages. (**I**) bud stage; (**II**) display-petal stage; (**III**) initial-flowering stage; (**IV**) full-flowering stage; (**V**) end-flowering stage; (**VI**) wilted-flower stage; and (**VII**) dried flower stage.

**Figure 2:**
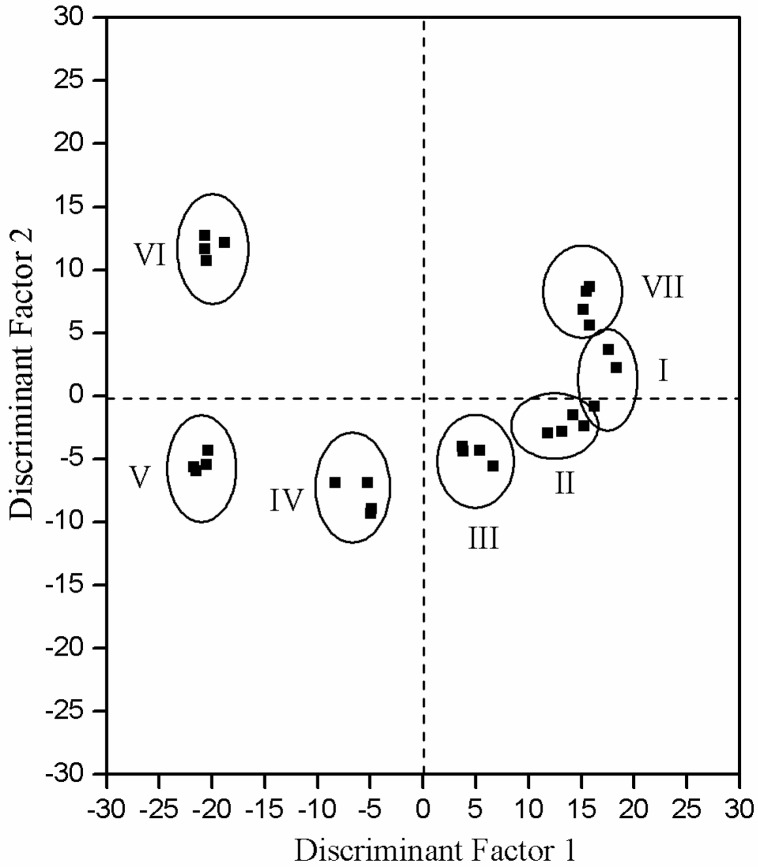
Two-dimensional (2D) DFA plots of flower from different stages by electronic nose. (**I**) bud stage; (**II**) display-petal stage; (**III**) initial-flowering stage; (**IV**) full-flowering stage; (**V**) end-flowering stage; (**VI**) wilted-flower stage; and (**VII**) dried flower stage.

*C. odorata* flowers were selected on the basis of their botanical characteristics to evaluate floral volatile polymorphisms according to different development stages: bud, display-petal, initial-flowering, full-flowering, end-flowering, wilted-flower, and dried flower stages [12] (Figure 1). To discriminate the different flower stages better, an electronic nose was coupled with discriminant factor analysis (DFA) [13,14] to classify the aroma emitted by *C. odorata* flowers. The process was conducted by using signals corresponding to four repeated exposures of each stage, as shown in Figure 2. The DFA score plot in Figure 2 shows that the electronic nose effectively discriminates each of the different flower stages of *C. odorata*. The first two discriminant factors, DF1 (81.16%) and DF2 (16.36%), explain 97.52% of total system variance. DFA is as valid as the multivariate statistical model if the percentage of recognition is higher than 90% [14]. In this case, the percentage of recognition was 97.52% (maximum 100%), which indicates that a certain degree of discrimination was achieved. For instance, the initial-flowering, full-flowering, and end-flowering stages were clearly distinguishable from one group to another, although a small overlap was observed between the bud and display-petal stages. These results show that the odors emitted at different stages of *C. odorata* flower development evidently differed. As Wilson and Baietto [13] suggested, electronic nose analysis based on botanical characteristics is a useful technique to discriminate flower aromas at different stages.

### 2.2. Analysis of C. odorata Floral Volatile Polymorphism by Headspace-Solid Phase Microextraction-Gas Chromatography-Mass Spectrometry (HS-SPME-GC-MS)

**Figure 3:**
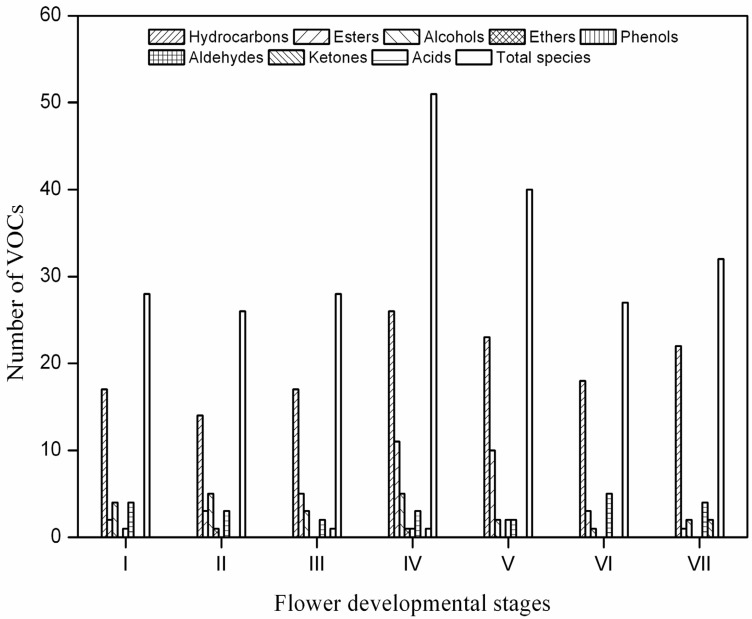
The composition of volatile compounds in different stages of *C. odorata* flower development by HS-SPME-GC-MS. (**I**) bud stage; (**II**) display-petal stage; (**III**) initial-flowering stage; (**IV**) full-flowering stage; (**V**) end-flowering stage; (**VI**) wilted-flower stage; and (**VII**) dried flower stage.

Previous researchers [7,8,9,10] have extensively investigated the volatile compositions of essential oils from *C. odorata* flower by using the hydrodistillation method [10,15]. However, distillation has several disadvantages, including time consumption, and loss of target compounds because of thermal degradation [16,17,18,19,20]. Studies on the volatile compounds present at different stages of *C. odorata* flower development have never been conducted elsewhere. Therefore, the volatile compounds in *C. odorata* flowers were analyzed by using HS-SPME coupled with GC-MS. Table 1 shows the 92 components identified over the flower-life. These components include 47 hydrocarbons, 17 esters, 14 alcohols, seven aldehydes, two phenols, 1 acid, three ketones, and one ether. In accordance with the report by Stashenko *et al.* [9], Burdock and Carabin [3], the total profile of the volatile compounds in all studied stages reveals the predominance of hydrocarbons, esters, alcohols, and aldehydes (Figure 3). Of these 92 compounds, 11 were particularly identified in the full-flowering stage on the basis of 15 AFNOR (ISO 3063: 2004 (E)). This finding is considered as characteristic of ylang-ylang essential oil. Additionally, volatile compositions varied considerably among the seven life-flower stages of *C. odorata* flowers. The mean Bray–Curtis similarity (*BCS*) value was 52.45% ± 11.61% (range: 32.84%–77.34%, *n* = 21 comparisons, Table 2). The full-flowering stage was more similar to the initial-flowering stage (*BCS* = 59.68%) than to the dried flower stage (*BCS* = 42.63%), and was largely dissimilar to the wilted-flower stage (*BCS* = 35.02%). As regards the comparison among the studied stages, 10 volatiles among the total volatile constituents notably existed in all life-flower stages, whereas 11 more volatile compounds were present in four of the seven stages. The results showed low similarity among the seven stages, although several constitutes [α-cadinol, farnesol isomer *a*, nerolidol, α-pinene, *etc.*] were exclusively identified in different stages. The pattern may indicate a relatively high volatile diversity variation at a full life-flower scale.

**Table 1:** Volatile compounds identified in seven different stages of *C. odorata* flower development using HS-SPME-GC-MS. (I) bud stage; (II) display-petal stage; (III) initial-flowering stage; (IV) full-flowering stage; (V) end-flowering stage; (VI) wilted-flower stage; and (VII) dried flower stage.

Peak	*RT* * (min.)	*LRI* *	Compounds	Relative Content (%) ± SD						
				I	II	III	IV	V	IV	VII
**Acids**										
1	9.001	1020	(*S*)-*α*-methoxybenzeneacetic acid	-	-	6.631 ± 0.601	11.711 ± 0.571	-	-	-
**Esters**										
2	8.460	1002	(*Z*)-3-hexen-1-ol acetate	-	-	-	0.070 ± 0.012	-	-	-
3	8.675	1009	hexyl acetate	-	-	-	0.026 ± 0.001	-	-	-
4	8.746	1011	(*E*)-2-hexen-1-ol acetate	-	-	-	0.044 ± 0.019	-	-	-
5	11.219	1094	methyl benzoate	-	0.067 ± 0.011	0.308 ± 0.002	2.605 ± 0.673	5.604 ± 0.129	-	-
6	13.240	1162	benzyl acetate	-	-	-	0.149 ± 0.001	0.126 ± 0.030	-	-
7	13.495	1170	benzoic acid, ethyl ester	-	-	-	0.314 ± 0.260	0.226 ± 0.085	-	-
8	15.481	1238	linalyl acetate	-	-	-	-	-	0.139 ± 0.380	-
9	17.191	1298	methyl 2-methoxybenzoate	-	-	0.050 ± 0.012	-	-	-	-
10	18.810	1358	lavandulyl acetate	-	-	-	0.079 ± 0.016	0.063 ± 0.014	-	-
11	19.258	1374	1,2-ethanediol 1-benzoate	-	-	-	0.304 ± 0.239	-	-	-
12	19.369	1378	neryl acetate	-	-	7.882 ± 0.227	11.740 ± 0.718	-	-	-
13	19.380	1379	geraniol acetate	-	0.075 ± 0.051	-	-	13.777 ± 1.311	-	-
14	21.092	1445	cinnamyl acetate	-	-	-	0.073 ± 0.008	0.161 ± 0.015	-	-
15	24.231	1572	(*Z*)-3-hexenyl benzoate	0.123 ± 0.013	-	0.115 ± 0.042	-	0.118 ± 0.014	0.144 ± 0.001	-
16	28.647	1768	benzyl benzoate	1.638 ± 0.054	2.498 ± 0.004	2.239 ± 0.802	2.146 ± 1.489	6.150 ± 0.896	2.893 ± 0.817	0.668 ± 0.226
17	30.801	1871	benzyl salicylate	-	-	-	0.066 ± 0.014	0.113 ± 0.030	-	-
18	32.626	1963	geranyl benzoate	-	-	-	-	0.044 ± 0.018	-	-
**Alcohols**										
19	4.919	854	3-hexen-1-ol	-	0.086 ± 0.025	-	-	-	-	-
20	9.349	1031	α-toluenol	-	-	-	0.071 ± 0.048	0.086 ± 0.033	-	-
21	9.365	1032	(±)-1,2-ethanediol, 1,2-diphenyl-, (R*, R*)	0.159 ± 0.017	-	-	-	-	-	-
22	9.425	1034	*p*-tolualcohol	-	-	-	0.061 ± 0.006	-	-	-
23	11.364	1099	*β*-linalool	-	-	0.053 ± 0.010	0.062 ± 0.001	-	-	-
24	15.819	1250	*β*-geraniol	1.396 ± 0.101	2.262 ± 0.102	-	-	-	-	-
25	23.698	1550	elemol	0.310 ± 0.045	-	-	-	-	-	-
26	24.402	1579	germacrene *D*-4-ol	-	0.387 ± 0.106	0.335 ± 0.042	-	-	-	-
27	25.815	1640	10,10-dimethyl-2,6-dimethylenebicyclo[7.2.0]undecan-5-*β*-ol	-	-	-	-	-	-	0.425 ± 0.108
28	25.939	1646	*t*-cadinol	-	-	-	0.090 ± 0.010	-	-	-
29	26.214	1658	*α*-cadinol	-	0.146 ± 0.049	-	-	-	-	-
30	27.500	1715	geranylgeraniol	-	-	-	0.736 ± 0.129	-	0.027 ± 0.000	-
31	27.527	1716	farnesyl alcohol	0.406 ± 0.043	1.238 ± 0.038	0.383 ± 0.000	-	1.227 ± 0.319	-	-
32	29.988	1832	(*E*)-nerolidol	-	-	-	-	-	-	0.113 ± 0.000
**Hydrocarbons**										
33	6.558	931	*α*-pinene	0.196 ± 0.009	-	-	-	-	-	-
34	8.034	986	bicyclo[3.1.1]hept-2-ene, 3,6,6-trimethyl	-	-	-	0.138 ± 0.025	-	-	-
35	8.057	987	*β*-pinene	0.132 ± 0.024	-	4.061 ± 0.000	2.861 ± 0.277	6.872 ± 1.055	-	-
36	8.067	988	*β*-myrcene	-	0.106 ± 0.005	0.118 ± 0.010	-	-	-	0.097 ± 0.000
37	9.262	1029	*α*-limonene	-	-	-	0.078 ± 0.009	-	0.029 ± 0.008	0.030 ± 0.000
38	9.283	1030	3-ethylidenecycloheptene	-	-	-	-	0.072 ± 0.012	-	-
39	10.961	1086	isoterpinolene	-	-	-	0.022 ± 0.003	-	-	-
40	19.348	1376	*α*-ylangene	4.093 ± 0.099	-	-	-	-	-	-
41	19.357	1378	*α*-copaene	-	6.019 ± 0.361	-	-	-	2.669 ± 0.298	-
42	19.516	1384	*p*-anisyl acetate	-	-	-	-	0.107 ± 0.028	-	-
43	19.522	1384	cyclohexene, 1-methyl-5-(1-methylethenyl)	-	-	-	0.058 ± 0.009	-	-	-
44	19.587	1386	*α*-bourbonene	-	-	-	-	-	-	0.118 ± 0.000
45	19.715	1391	*β*-elemen	1.545 ± 0.076	-	-	0.736 ± 0.088	-	0.648 ± 0.228	-
46	20.591	1425	*β*-caryophyllene	33.296 ± 0.710	32.526 ± 0.456	26.866 ± 1.198	17.013 ± 0.233	15.048 ± 1.629	21.154 ± 0.230	27.904 ± 0.462
47	20.789	1432	*β*-cubebene	0.382 ± 0.022	7.139 ± 0.617	7.347 ± 0.619	11.790 ± 1.428	12.914 ± 1.285	0.691 ± 0.052	10.012 ± 0.883
48	21.014	1439	*α*-selinene	-	-	-	-	0.025 ± 0.005	-	-
49	21.015	1441	*γ*-caryophyllene	-	-	-	-	-	-	0.038 ± 0.002
50	21.021	1442	*L*-alloaromadendrene	-	-	0.037 ± 0.000	12.792 ± 1.004	-	-	-
51	21.179	1448	isoledene	-	-	-	0.088 ± 0.001	-	-	-
52	21.288	1452	*α*-cubebene	1.095 ± 0.173	0.448 ± 0.196	0.417 ± 0.036	0.147 ± 0.015	0.183 ± 0.019	0.159 ± 0.016	0.574 ± 0.167
53	21.312	1453	(*E*)-*β*-farnesene	-	-	-	0.284 ± 0.243	0.522 ± 0.040	0.931 ± 0.000	0.895 ± 0.000
54	21.473	1460	*α*-caryophyllene	13.975 ± 0.545	12.974 ± 0.157	10.189 ± 0.042	5.966 ± 0.416	5.220 ± 0.598	8.366 ± 0.017	9.037 ± 0.228
55	22.106	1485	*D*-germacrene	10.857 ± 0.191	13.732 ± 0.000	7.018 ± 0.627	1.067 ± 0.008	0.462 ± 0.053	19.498 ± 1.054	9.027 ± 0.792
56	22.366	1495	(+)-epi-bicyclosesquiphellandrene	1.457 ± 0.213	0.592 ± 0.422	-	0.320 ± 0.022	0.327 ± 0.070	-	-
57	22.449	1498	bicyclogermacrene	1.767 ± 0.593	1.994 ± 0.386	0.752 ± 0.030	0.511 ± 0.051	0.538 ± 0.078	0.723 ± 0.079	0.418 ± 0.000
58	22.500	1500	*α*-muurolene	-	0.522 ± 0.000	0.516 ± 0.000	0.576 ± 0.072	0.274 ± 0.012	-	-
59	22.589	1501	(*Z*,*E*)-*α*-farnesene	5.144 ± 0.249	-	-	6.913 ± 1.857	-	-	-
60	22.602	1505	[*S*-(R*,S*)]-5-(1,5-dimethylhexen-4-yl)-2-methyl-1,3-cyclohexa-1,3-diene	-	-	-	-	-	-	14.114 ± 0.000
61	22.639	1507	*α*-farnesene	-	-	11.185 ± 0.000	0.070 ± 0.001	5.935 ± 0.306	15.687 ± 0.000	0.117 ± 0.025
62	22.793	1512	(−)-*β*-bisabolene	-	-	-	0.196 ± 0.113	0.267 ± 0.050	0.570 ± 0.000	0.423 ± 0.000
63	22.931	1518	bicyclo[4.4.0]dec-1-ene 2-isopropyl-5-methyl-9-methylene	-	0.751 ± 0.000	-	-	-	-	0.768 ± 0.000
64	22.982	1520	*δ*-cadinene	3.361 ± 0.118	2.426 ± 0.394	2.405 ± 0.087	2.405 ± 0.117	1.315 ± 0.112	1.723 ± 0.186	2.132 ± 0.005
65	23.067	1524	l-calamenene	1.078 ± 0.075	-	-	-	-	-	0.726 ± 0.000
66	23.101	1525	4,9-cadinadiene	-	-	-	-	0.271 ± 0.027	-	0.311 ± 0.000
67	23.176	1528	*cis*-*α*-bisabolene	-	-	-	-	0.081 ± 0.005	-	-
68	23.333	1534	*α*-cedrene	-	-	-	-	0.111 ± 0.011	0.163 ± 0.000	0.199 ± 0.006
69	23.334	1535	1,4-cadinadiene	-	0.133 ± 0.030	0.568 ± 0.014	-	-	-	-
70	23.335	1536	*α*-funebrene	-	-	-	0.189 ± 0.039	-	-	-
71	23.434	1538	bicyclo[2.2.1]heptane 2-cyclopropylidene-1,7,7-trimethyl	-	-	-	0.440 ± 0.102	0.102 ± 0.005	0.272 ± 0.149	-
72	23.697	1550	(*E*,*Z*)-*α*-farnesene	-	-	0.036 ± 0.019	-	-	0.040 ± 0.000	-
73	23.818	1554	*α*-bergamotene	-	-	0.061 ± 0.030	-	-	-	-
74	23.819	1555	*β*-gurjurene	1.277 ± 0.165	0.036 ± 0.001	0.131 ± 0.000	1.159 ± 0.041	0.131 ± 0.001	0.531 ± 0.247	0.939 ± 0.249
75	24.401	1578	*β*-bourbonene	-	-	-	0.125 ± 0.009	-	-	-
76	24.548	1585	*β*-caryophyllene oxide	-	-	-	0.042 ± 0.015	-	-	3.659 ± 0.365
77	25.590	1630	(+)-*α*-longipinene	0.123 ± 0.013	-	-	-	-	-	-
78	25.937	1646	copaene	0.229 ± 0.028	-	0.069 ± 0.000	0.179 ± 0.117	0.297 ± 0.232	0.434 ± 0.060	3.339 ± 0.206
79	26.210	1659	elixene	-	-	-	-	0.247 ± 0.212	-	-
**Phenols**										
80	10.516	1071	*p*-kresol	-	-	-	0.174 ± 0.107	0.048 ± 0.013	-	-
81	19.918	1399	1,2-dimethoxy-4-(2-propenyl)-benzen	0.087 ± 0.020	-	-	-	0.138 ± 0.056	-	-
**Ethers**										
82	14.346	1099	estragole	-	0.137 ± 0.008	-	0.125 ± 0.009	-	-	-
**Aldehydes**										
83	7.313	959	benzaldehyde	0.142 ± 0.014	0.072 ± 0.021	-	-	-	4.193 ± 0.413	1.600 ± 0.000
84	9.684	1043	benzeneacetaldehyde	-	-	-	-	-	0.177 ± 0.094	-
85	15.479	1237	citral *b*	-	-	0.202 ± 0.000	-	0.059 ± 0.006	-	0.538 ± 0.000
86	15.486	1239	*β*-citral	0.623 ± 0.111	0.512 ± 0.512	-	0.194 ± 0.086	-	-	0.081 ± 0.025
87	16.322	1268	*α*-citral	2.226 ± 0.269	2.166 ± 0.221	1.756 ±0.085	1.685 ± 0.314	1.377 ± 0.046	1.021 ± 0.093	1.009 ± 0.005
88	27.365	1709	farnesal	-	-	-	-	-	1.128 ± 0.014	-
89	27.965	1736	(*E*,*E*)-farnesal	0.192 ± 0.014	-	-	0.034 ± 0.023	-	3.131 ± 1.136	-
**Ketones**										
90	7.892	981	methyl heptenone	-	-	-	-	-	-	0.218 ± 0.013
91	14.153	1192	2-hydroxy-3-cyanopyridine	-	-	-	0.047 ± 0.001	0.101 ± 0.055	-	0.089 ± 0.007
92	25.197	1613	junenol	-	-	-	-	-	-	0.665 ± 0.000

* *RT* = Retention time (minutes); * *LRI* = Linear retention indices to C_7_-C_30_ *n*-alkanes on DB-5MS column, *LRI* literatures were according to the results of *Benini et al.* [2], the database of *VCF* 15.1, and the *Pherobase*.

**Table 2:** The Bray-Curtis similarity values (%) among different stages of *C. odorata* flower development. (I) bud stage; (II) display-petal stage; (III) initial-flowering stage; (IV) full-flowering stage; (V) end-flowering stage; (VI) wilted-flower stage; and (VII) dried flower stage.

	I	II	III	IV	V	VI	VII
I	100						
II	77.34	100					
III	58.37	66.78	100				
IV	41.61	41.90	59.68	100			
V	32.84	42.16	52.26	50.98	100		
VI	54.90	60.00	61.08	35.02	41.49	100	
VII	59.99	65.60	61.02	42.63	42.33	53.44	100

**Figure 4:**
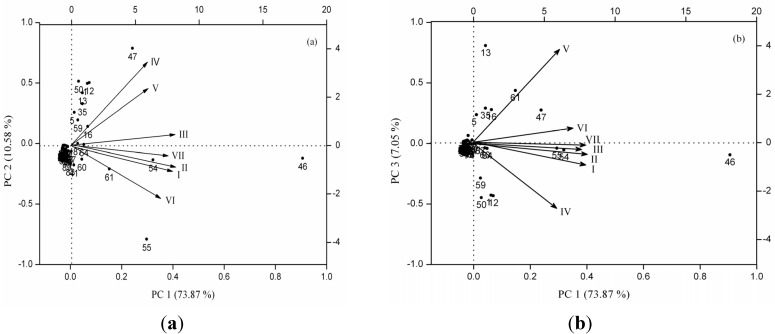
Principal components analysis biplot showing relationship between the different stages of *C. odorata* flower development and the volatile compounds: PC1 *vs.* PC2 plots (**a**) and PC 1 *vs.* PC 3 plots (**b**). (I) bud stage, (II) display-petal stage, (III) initial-flowering stage, (IV) full-flowering stage, (V) end-flowering stage, (VI) wilted-flower stage, and (VII) dried flower stage. Black dots represent distribution of 92 volatile compounds in *C. odorata* flower (numbers correspond to those in Table 1).

Variations of the volatile compositions emitted by *C. odorata* flowers significantly depend on the stage of maturity. The same phenomena are also observed in other plant species, such as *Michelia alba* [21], and *Michelia champaca* flowers [16]. In this study, the highest floral volatile polymorphism was detected at the intermediate levels of flower development, and the richness of volatile compounds showed a unimodal or hump-shaped pattern between the number of VOCs and times of flower development. To identify which volatiles contributed the most to the differences among the seven flower stages, the data on 92 volatile compounds identified in *C. odorata* at a full life-flower scale were analyzed by using principal component analysis (PCA). The first three components of PCA explained 73.87%, 10.58%, and 7.05% of the variation, explaining ~92% of combined variance (Figure 4). Hereinto, volatiles that had high positive scores on PC 1 include α-caryophyllene, d-germacrene, α-farnesene, δ-cadinene, α-citral, and β-caryophyllene, which are highly positively related to the bud, display-petal, and initial-flowering stages. Volatiles with high positive scores on PC 2 include benzyl benzoate, methyl benzoate, (*S*)-α-methoxybenzeneacetic acid, neryl acetate, geraniol acetate, β-pinene, β-cubebene, (*Z*,*E*)-α-farnesene, and l-alloaromadendrene, which are negatively correlated with the wilted-flower stage. The remaining 77 volatiles components, which include common components, α-cubebene, bicyclogermacrene, β-gurjurene, and copaene, as well as relatively rare volatile compounds, including 3-hexen-1-ol, β-myrcene, α-ylangene, do not exhibit any association with the first three components.

As for the bud stage, 28 volatile compounds belonging to different chemical classes were identified: hydrocarbons (80.01%), esters (1.76%), aldehydes (3.18%), and alcohols (2.27%). The most abundant compound was β-caryophyllene, accounting for about 35% of the total GC peak area, followed by α-caryophyllene (13.98%), d-germacrene (10.86%), (*Z*,*E*)-α-farnesene (5.14%), and α-ylangene (4.09%). With respect to other flower stages, the bud stage was characterized by higher hydrocarbons.

As for the display-petal stage, 26 volatile compounds belonging to different chemical classes were identified: hydrocarbons (79.40%), esters (2.64%), alcohols (4.12%), and aldehydes (2.75%). The most abundant compound was β-caryophyllene, accounting for about 32% of the total GC peak area, followed by d-germacrene (13.73%), α-caryophyllene (12.97%), β-cubebene (7.14%), and *α*-copaene (6.02%). With respect to other flower stages, the display-petal stage was characterized by higher alcohols.

As for the initial-flowering stage, 28 volatile compounds belonging to different chemical classes were identified: hydrocarbons (71.78%), esters (10.59%), acids (6.63%), alcohols (0.77%), and aldehydes (1.96%). The most abundant compound was β-caryophyllene, accounting for about 26.87% of the total GC peak area, followed by α-farnesene (11.86%), α-caryophyllene (10.19%), neryl acetate (7.89%), β-cubebene (7.35%), and d-germacrene (7.02%). With respect to other flower stages, the initial-flowering stage was characterized by higher acids.

As for the full-flowering stage, 51 volatile compounds belonging to different chemical classes were identified: hydrocarbons (66.17%), esters (17.62%), acides (11.72%), aldehydes (3.83%), and alcohols (1.02%). The most abundant compound was β-caryophyllene, accounting for about 17.03% of the total GC peak area, followed by l-alloaromadendrene (12.79%), β-cubebene (11.79%), neryl acetate (11.74%), (*S*)-α-methoxybenzeneacetic acid (11.71%), and (*Z*,*E*)-α-farnesene (6.91%). With respect to other flower stages, the full-flowering stage was characterized by higher esters and acids.

As for the end-flowering stage, 40 volatile compounds belonging to different chemical classes were identified: hydrocarbons (51.07%), esters (26.38%), alcohols (1.13%), and aldehydes (2.87%). The most abundant compound was β-caryophyllene, accounting for about 15.05% of the total GC peak area, followed by geraniol acetate (13.78%), β-cubebene (12.91%), β-pinene (6.87%), benzyl benzoate (6.15%), and α-farnesene (5.94%). With respect to other flower stages, the end-flowering stage was characterized by higher esters.

As for the wilted-flower stage, 27 volatile compounds belonging to different chemical classes were identified: hydrocarbons (74.29%), aldehydes (19.30%), and esters (3.18%). The most abundant compound was β-caryophyllene, accounting for about 21.15% of the total GC peak area, followed by d-germacrene (19.50%), β-cubebene (0.69%), α-farnesene (15.69%), α-caryophyllene (8.37%), and benzaldehyde (4.19%). With respect to other flower stages, the wilted-flower stage was characterized by higher aldehydes.

As for the dried flower stage, 32 volatile compounds belonging to different chemical classes were identified: hydrocarbons (84.88%), aldehydes (6.46%), and esters (0.69%). The most abundant compound was β-caryophyllene, accounting for about 27.90% of the total GC peak area, followed by [*S*-(R*,S*)]-5-(1,5-dimethylhexen-4-yl)-2-methyl-1,3-cyclohexa-1,3-diene (14.11%), β-cubebene (10.01%), α-caryophyllene (9.04%), and d-germacrene (9.03%). With respect to other flower stages, the dried flower stage was characterized by higher hydrocarbons.

## 3. Experimental

### 3.1. Plant Materials

Fresh *C. odorata* flowers came from populations grown at the Spice and Beverage Research Institute, CATAS in Hainan, China. The flowers were classified into seven groups according to their botanical characteristics (Figure 1): (I) bud stage: buds emerged from scaly bracts, yellowish green, completely closed; (II) display-petal stage: completely open calyxes, petals spreading, pubescent, and green; (III) initial-flowering stage: semi-open petals, light green; (IV) full-flowering stage: completely open petals, green, observable pistils and stamens; (V) end-flowering stage: fully matured petals of deep yellow coloration; (VI) wilted-flower stage: petals and calyxes withered and yellow; and (VII) dried- flower stage: petals dried.

### 3.2. Methods

The inflorescence of *C. odorata* is a raceme that always exhibits inconsistent flowering [12]. Approximately 20 g of raw flower material of *C. odorata* was collected at 8:00 a.m.–10:00 a.m., 8–10 August 2013, depending on the different stages of flower development. Following collections, the flowers were moisturized and sent back to the laboratory immediately. In all experiments, flowers were sliced by using a knife into similarly thin slices to be able to place the sample in a headspace bottle (volume ~5 mL) while maintaining uniform flower sample structure and weight. Thereafter, 1.5 g of sliced materials were weighted and allowed to stand for 30 min at ambient room temperature 22 ± 3 °C. The assayed fibers used in this study were DVB-CAR-PDMS with a 50/30 μm film thickness (Supelco, Bellefonte, PA, USA). The SPME device was inserted into the sealed vial by manually penetrating the silicone septum, and the fiber was exposed to the headspace of the sliced material after 40 min. After extraction, the needle on the SPME manual holder was set to 0.5 cm in the GC injector. The fiber was then directly desorbed for 10min. Each sampling and analysis step was performed in triplicate. Empty bottles were used as a control in the analyses.

An HP-7890A/5975C GC-MS system (Agilent Technologies, Wilmington, USA), with a DB-5MS column (30 m × 0.25 mm I.D. × 0.25 μm microns, Agilent Technologies) was used under the following conditions: MS transfer line heater of 280 °C, injector temperature of 250 °C, and operation in the splitless mode. Initial oven temperature was held at 50 °C for 5 min., then programmed from 50 °C to 80 °C at 10 °C/min, from 80 °C to 220 °C at 5 °C/min, from 220 °C to 280 °C at 10 °C/min, and finally maintained for 6 min at 280 °C. Helium gas was used as a carrier gas at a flow rate of 1.0 mL/min. An Agilent 5975 C mass spectrometer was operated in the electron ionization mode at 70 eV with a source temperature of 230 °C, a quadrupole set to 150 °C, and a scan from an *m*/*z* 30 to 500 in the full-scan mode.

Linear retention indices (*LRI*) were determined on the basis of alkanes series (C_7_–C_30_). It is defined as:

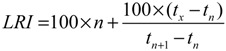
(1)
where *t_n_* and *t_n+_*_1_ are retention times of the reference n-alkanes hydrocarbons eluting immediately before and after chemical compound “*x*”, *t_x_* is the retention time of compound “*x*”. Volatile compounds were identified on the basis of their *LRI* and by comparing their mass spectra with a computerized MS-database using NIST 2008 library, Volatile Compounds in Food 15.1, and the Pherobase (database of pheromones and semi-chemicals). We also compared the fragmentation patterns in the mass spectra with those reported in the literatures [2,9]. The concentrations of the component (relative contents were done by calculating percentage of peak area in GC chromatograms), computerized by normalization method from the equation:
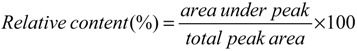
(2)

Additionally, a Bray-Curtis similarity (BCS), signifying the compositional similarity different flower developmental stages, was used to examine whether the floral volatiles in *C. odorata* were different. This index incorporates the presence as well as relative content of the component [22] It is defined as:

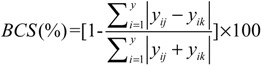
(3)
where y*_ij_* and y*_ik_* are the relative content of component *_i_* in group *_j_* and *_k_*, respectively, and *_p_* is the total number of components in both groups.

### 3.3. Data Analysis

Volatile compounds identified in *C. odorata* at a full life-flower scale were analyzed by using principal component analysis (PCA). PCA was carried out using R package version 2.12.2 for Windows.

## 4. Conclusions

The odors emitted by *C. odorata* flowers evidently differed among flower development stages. Ninety-two volatile compounds were identified in all flower-life stages, and the main compounds were caryophyllene, α-caryophyllene, β-cubebene, d-germacrene, benzyl benzoate, δ-cadinene, β-gurjurene, α-citral, and α-cubebene. The relative content of benzyl benzoate and β-cubebene became more obvious during the expansion of bud and full opening of flowers. However, when the flower developed further, the emissions of these major components decreased, whereas those of β-caryophyllene and d-germacrene increased. Moreover, the volatile compounds emitted by *C. odorata* flowers largely depend on the stage of maturity. Large numbers of VOCs emerged at intermediate times of flower development. More hydrocarbon, esters, and alcohols compounds were detected in the full-flowering stage than in other flower stages, which may have contributed to the aroma profile of the flowers. Thus, a high level of floral volatile polymorphism was observed in *C. odorata* at a full life-flower scale. Under these circumstances, measures that introduce timely harvest, such as selective harvesting at intermediate levels of flower development, are recommended when the high level of volatile compounds and the aroma quality of flowers are a concern.

## References

[1] Kristiawan M., Sobolik V., Allaf K. (2008). Isolation of Indonesian cananga oil using multi-cycle pressure drop process. J. Chromatogr. A.

[2] Benini C., Ringuet M., Wathelet J.P., Lognay G., du Jardin P., Fauconnier M.L. (2012). Variations in the essential oils from ylang-ylang (*Cananga odorata* [Lam.] Hook f. & Thomson forma *genuina*) in the Western Indian Ocean islands. Flavour Frag. J..

[3] Burdock G.A., Carabin L.G. (2008). Safety assessment of ylang-ylang oil as a food ingredient. Food Chem. Toxicol..

[4] Jung D.J., Cha J.Y., Kim S.E., Ko I.G., Jee Y.S. (2013). Effect of ylang-ylang aroma on blood pressure and heart rate in healthy men. J. Exerc. Rehabil..

[5] Benini C., Danflous J.P., Wathelet J.P., du Jardin P., Fauconnier M.L. (2010). Ylang-ylang [*Cananga odorata* (Lam.) Hook. f. & Thomson]: An unknown essential oil plant in an endangered sector. Biotechnol. Agron. Soc..

[6] Kristiawan M., Sobolik V., Allaf K. (2012). Yield and composition of Indonesian cananga oil obtained by steam distillation and organic solvent extraction. Int. J. Food Eng..

[7] Brokl M., Fauconnier M.L., Benini C., Lognay G., du Jardin P., Focant J.F. (2013). Improvement of Ylang-Ylang essential oil characterization by GC-GC-TOFMS. Molecules.

[8] Gaydou E.M., Randriamiharisoa R., Bianchini J.P. (1986). Composition of the essential oil of Ylang-Ylang (*Cananga odorata* Hook Fil. & Thomson forma *genuina*) from Madagascar. J. Agric. Food Chem..

[9] Stashenko E.E., Torres W., Morales J.R.M. (1995). A study of the compositional variation of the essential oil of ylang-ylang (*Cananga odorata* Hook Fil. & Thomson, forma *genuina*) during flower development. J. High Resolut. Chromatogr..

[10] Benini C., Mahy G., Bizoux J.P., Wathelet J.P., du Jardin P., Brostaux Y., Fauconnier M.L. (2012). Comparative chemical and molecular variability of *Cananga odorata* (Lam.) Hook. f. & Thomson forma genuina (Ylang-Ylang) in the Western Indian Ocean islands: Implication for valorization. Chem. Biodivers..

[11] AFNOR (2005). Oil of Ylang-Ylang [Cananga odorata (Lam.) Hook. f. & Thomson forma genuina].

[12] Editorial Committee of Flora Reipublicae Popularis Sinicae (1979). Flora Reipublicae Popularis Sinicae.

[13] Wilson A.D., Baietto M. (2011). Advances in electronic-nose technologies development for *biomedical applications*. Sensors.

[14] Zhang M.X., Wang X.C., Liu Y., Xu X.L., Zhou G.H. (2012). Species discrimination among three kinds of puffer fish using an electronic nose combined with olfactory sensory evaluation. Sensors.

[15] Olivero J., Gracia T., Payares P., Vivas R., Diaz D., Daza E., Paul G. (1997). Molecular structure and gas chromatographic retention behavior of the components of Ylang-Ylang oil. J. Pharm. Sci..

[16] Jiang D.Y., Li Y.H., He F., Lin Q.P., Pan H.T. (2012). The components and changes of VOCs of *Michelia champaca* L. flower at different developmental stages. Sci. Agric. Sin..

[17] Agah M., Najafian S. (2012). Essential oil content and composition of *Lippa citriodora* as affected by drying method before flowering stages. Eur. J. Exp. Biol..

[18] Chen H.C., Chi H.S., Lin L.Y. (2013). Headspace solid-phase microextraction analysis of volatile components in *Narcissus tazetta* var. *chinensis* Roem. Molecules.

[19] Li L., Zhao J.C. (2009). Determination of the volatile composition of *Rhodobryum giganteum* (Schwaegr.) Par. (Bryaceae) using solid-phase microextraction and gas chromatography/mass spectrometry (GC/MS). Molecules.

[20] Li J., Liu X.G., Dong F.S., Xu J., Zheng Y.Q., Shan W.L. (2010). Determination of the volatile composition in essential oil of *Descurainia sophia* (L.) Webb ex Prantl (Flixweed) by gas chromatography/mass spectrometry (GC/MS). Molecules.

[21] Sanimah S., Suri R., Azizun R.N., Hazniza A., Radzali M., Rusli I., Hassan M.D. (2008). Volatile compounds of essential oil from different stages of *Michelia alba* (cempaka putih) flower development. J. Trop. Agric. Food Sci..

[22] Clarke K.R. (1993). Non-parametric multivariate analyses of changes in community structure. Aust. J. Ecol..

